# A hierarchical model of metabolic machinery based on the *k*core decomposition of plant metabolic networks

**DOI:** 10.1371/journal.pone.0195843

**Published:** 2018-05-07

**Authors:** Humberto A. Filho, Jeaneth Machicao, Odemir M. Bruno

**Affiliations:** São Carlos Institute of Physics, University of São Paulo, São Carlos - SP, PO Box 369, 13560-970, Brazil; Universidad Nacional de Rosario, ARGENTINA

## Abstract

Modeling the basic structure of metabolic machinery is a challenge for modern biology. Some models based on complex networks have provided important information regarding this machinery. In this paper, we constructed metabolic networks of 17 plants covering unicellular organisms to more complex dicotyledonous plants. The metabolic networks were built based on the substrate-product model and a topological percolation was performed using the *k*core decomposition. The distribution of metabolites across the percolation layers showed correlations between the metabolic integration hierarchy and the network topology. We show that metabolites concentrated in the internal network (maximum *k*core) only comprise molecules of the primary basal metabolism. Moreover, we found a high proportion of a set of common metabolites, among the 17 plants, centered at the inner *k*core layers. Meanwhile, the metabolites recognized as participants in the secondary metabolism of plants are concentrated in the outermost layers of the network. This data suggests that the metabolites in the central layer form a basic molecular module in which the whole plant metabolism is anchored. The elements from this central core participate in almost all plant metabolic reactions, which suggests that plant metabolic networks follows a centralized topology.

## Introduction

It is believed that the general architecture of living systems is based on the structure and function of modules [1]. However, it is still a challenge for biology to understand how this modular organization is structured from the molecular to the cellular level. A model that relates how biological modules interact would be very useful. The metabolism of living beings has been understood as a network of interactions that has a modular organization [2]. At the same time the understanding of this metabolism as a complex network has brought new visions about the organization of the metabolic components and the network topology of possible interactions between the metabolites [3].

The metabolism is perhaps the best network of interactions ever characterized in biology because a large number of studies has defined metabolic pathways [4, 5]. For subsequent decades of enzymology applications, the catalytic and regulatory properties of enzymes have been characterized. More recently genetic studies and molecular biology have opened up avenues in the knowledge of enzyme genes that catalyze reactions of transformations of matter in living beings. Thus, there is an unprecedented amount of descriptive and mechanistic data on the behavior of metabolic phenomena that can be analyzed as complex networks of which relevant information about living systems can be obtained.

Data extracted from plant genomes make it possible to investigate correlations of the molecular backbone of living beings that are related to their adaptations to the environment [6–8]. Complete data from the genome of various plants have generated a set of metabolic reactions based on the genes of enzymes that can resume the metabolism of these organisms. The metabolism is divided into discrete pathways, however it operates as a highly integrated network. Therefore, metabolic networks can be built and the metabolism of various living beings is modeled and quantified based on network parameters [3].

However, it is not known exactly how a general design on the distribution of matter in metabolism can be developed based on the observation of the topology from metabolic networks. Consequently, in order to reveal functional, hierarchical and even phylogenetic information hidden in the network structure, new models need to be investigated. The content of plant metabolic networks reflects the species phylogeny, thus groups of plants that are evolutionary closer share a larger proportion of common metabolites. In this case, the specialized metabolism is responsible for the greatest differences; gene coding for specialized metabolic functions have proliferated to a much greater degree and by different mechanisms and display lineage-specific patterns of physical clustering within the genome [9]. Thus, understanding the connectivity pattern of secondary metabolites in the metabolic networks of plants should be fundamental to the overall understanding of plant metabolism.

Although some studies have shown that the hierarchical organization and modularity inside the metabolic network is related to the chemical classes of metabolites [2], these studies lack a holistic model concerning how the organization of modules is centralized. This comprehensive model would find general principles that govern the structure and function of modules.

For instance, some attempts have been made to find fully-connected parts of the metabolic networks [10]. Completely connected cores of networks, in general, have been obtained by the *k*core decomposition [11, 12]. It is a well-known algorithm that has been used in many contexts, mostly in biological networks, such as protein-protein interaction [13]. Through *k*core percolation, a network is pealed as a set of successively *k*cores (layers (*k*)) until a maximum core is achieved. Thus, small values of *k* define the periphery of the network and the innermost network core corresponds to a large *k*. This algorithm has been proposed to understand the network hierarchical organization by extracting highly interconnected parts and thus providing ways to find relationships within these substructures [14]. Therefore, the *k*cores can provide useful insights into the global network topology and also establish a hierarchy of connections from each network node [15].

In this study, we present a percolation analysis based on the *k*core decomposition of plant metabolic networks modeled according to the plant metabolic reaction dataset from 17 plants selected from the PlantCyc database [16]. The metabolic networks were built by using a well-known model “substrate-product network” [17], where metabolites (nodes) from substrates are linked to metabolites from products of the reactions. Thus, the metabolic network of 17 plants, namely: *Brachypodium distachyon* (BD), *Hordeum vulgare* (HV), *Oryza sativa japonica* (OSJ), *Panicum virgatum* (PV), *Setaria italica* (SI), *Sorghum bicolor* (SB), *Zea mays* (ZM); *Arabidopsis thaliana* (AT), *Brassica rapa pekinensis* (BRP), *Carica papaya* (CP), *Glycine max* (GM), *Manihot esculenta* (ME), *Populus trichocarpa* (PT), *Vitis vinifera* (VV); *Selaginella moellendorffii* (SM); *Physcomitrella patens* (PP) and *Chlamydomonas reinhardtii* (CR) were considered.

Our main objective is to demonstrate that a percolation through the *k*core decomposition of plant metabolic networks reveals information about the metabolic centralization and functional hierarchy of certain metabolite groups distributed across its layers.

This functional analysis shows that a high proportion of the metabolites from central layers of the networks contain the basic components from basal metabolism. In addition, we show that the metabolites found in the more centralized level of the networks integrate all plant metabolisms because they participate in the absolute majority of their metabolic reactions. As confirmed by previous studies that show that metabolic networks are very modularized [2], we also find that plant metabolic networks form modules. The metabolites contained in these modules connect to the metabolites found in the central core of the networks in a much larger proportion than the modules themselves, which suggests that the central core can form a kind of metabolic module of the metabolic networks and that there is sub-modularization in the networks as has already been suggested in previous studies [2]. In addition, we found that basal metabolism is distributed along the *k*core layers of the networks in an opposite way relative to the secondary and non-common metabolism between plants. The common metabolism between plants is completely centralized in the layers with greater connectivity and the metabolism secondary and uncommon between the plants is peripheral along the layers. Together these data show that the decomposition by a layering tool is useful for building hierarchical models of the metabolism organization and brings information that can be used in studies about the evolution of the metabolism.

## Results

### kcore percolation of plant metabolic networks

We applied the *k*core percolation algorithm to 17 plant metabolic networks whose set of metabolic reactions were obtained from the PlantCyc database [16]. The set of metabolic reactions from each plant was used to built metabolic networks following the substrate-product model [17], which is a model that establishes the correlation of distribution of matter in metabolisms through a network of connections related to the formation and decomposition of components [18].

**Fig 1:**
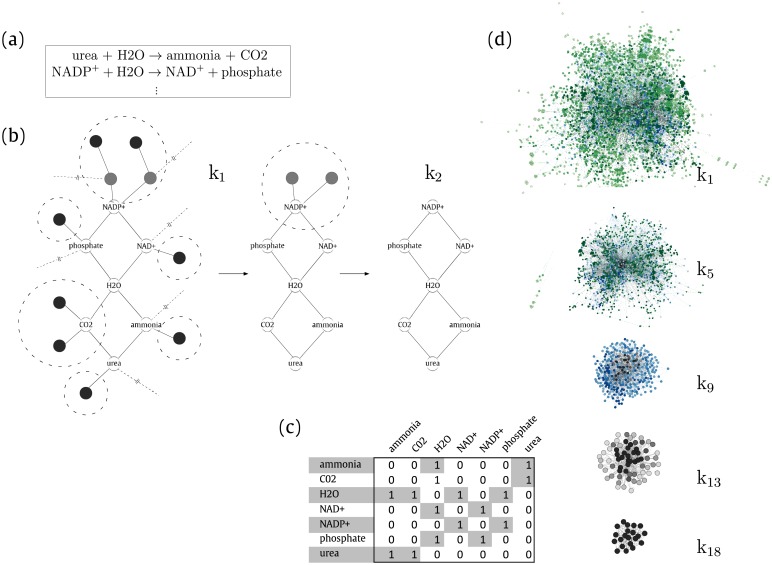
(a) Example of a set of simplified metabolic reactions that assemble a metabolic network, which is built considering each molecule of the reaction according to the substrate-product network model [17]. Thus, each substrate metabolite is individually linked to each one of the product metabolites and vice versa. (b) The *k*core decomposition of a given network. First iteration: the peripheral nodes with degree *k*_*i*_ = 1 (dark gray) are removed. Second iteration: The remaining network of the first iteration, which presents 9 nodes of which 2 of them contain only one edge (light gray), is pruned again. Finally, the resulting 2-core corresponds to a fully connected core composed of 7 nodes (white). (c) Resulting adjacency matrix corresponding to the 2-core shell where an outgoing link from the nodes in the first row of the matrix has an incoming link at each metabolite from the first column, and vice versa. (d) *k*core decomposition from *Arabidopsis thaliana* metabolic network. From top to down, the original network at *k*_1_ is pealed iteratively until *k*_max_ where it reaches *k* = 18 layers (see S7 File). Different colors were used to represent each layer. Edges are not shown.

The Fig 1a shows a given set of reactions from which a network is obtained. The *k*core decomposition is applied successively, as shown in Fig 1b, until a maximum core is obtained, whose corresponding adjacency matrix is shown in Fig 1c. The overall sketching of the distribution of layers in a generic metabolic network is shown in Fig 1d.

The *k*core percolation from the metabolic network of *Arabidopsis thaliana* shows that the last full connected level (maximum *k*core) contains 21 metabolites, while the outermost layer contains 3,546 metabolites, from which 149 belong exclusively to *k*_1_. As expected, there is a decrease in the number of metabolites in each layer until the central core. It can be observed that the maximum number of layers *k*_max_ for all the studied plants varies between 16 and 18. Thus, these plants were grouped into three sets: BD, HV, OSJ, SI and SB with 16 layers; PV, ZM, BRP, CP, GM, ME, VV, SM and CR with 17 layers; AT and PP with 18 layers.

We found that the number of layers *k*_max_ is not related to the number of metabolites (*N*). For instance, networks with relatively small sizes such as moss *Physicomitrela patens* (*N* = 2651) reached the highest number of cores (*k* = 18), while networks with larger sizes such as the dicotyledonous *Manihot esculenta* (*N* = 2991) reached fewer cores (*k* = 16). The detailed network properties, such as the number of layers and network size for the 17 plant metabolic networks studied here, can be found in S1 File.

**Fig 2:**
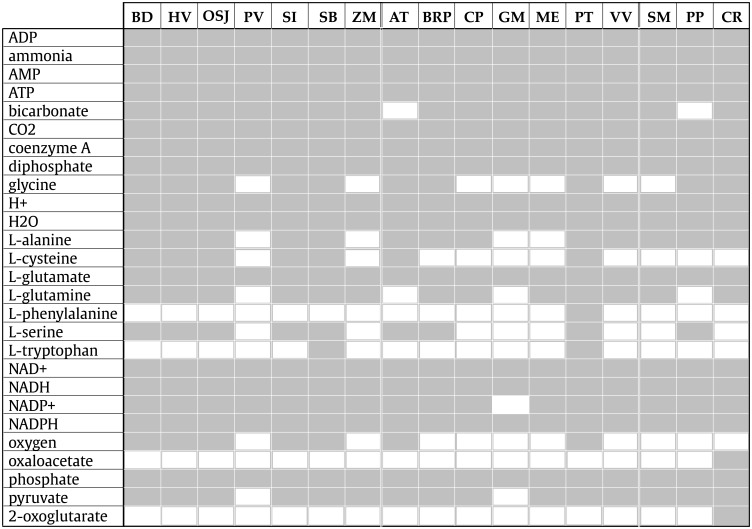
Metabolites found at the maximum core*k*_max_. The columns indicate the plant acronyms, while the metabolites are shown as rows. The white cells indicate the presence of a metabolite in a particular plant *k*core, while black cells indicate its absence.

We also analyzed the chemical composition of the central core in order to obtain the contents of the innermost network for the 17 plants, which is shown in Fig 2. It was observed that the central core of the plant networks only comprises molecules from the basal metabolism of plants. In this regard, 14 metabolites were found in common at the maximum *k*core from all plants studied here namely: ADP, ammonia, AMP, ATP, CO_2_, coenzyme A, diphosphate, H+, H_2_O, L-glutamate, NAD+, NADH, NADPH, and phosphate. These 14 metabolites extracted from the central core, found in the 17 studied plants, represent a very connected dataset.

### Integration of metabolism within the central *k*core

**Fig 3:**
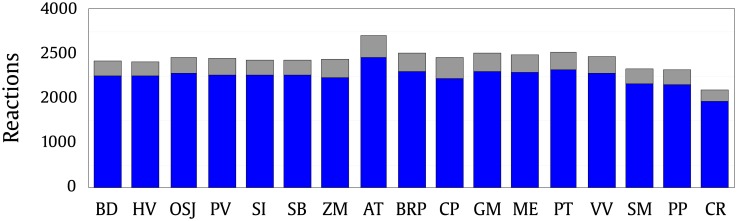
Plant reactions that contain at least one metabolite from the maximum *k*core shown by a blue bar, otherwise by a gray bar.

We observed that the metabolites extracted from the central core of the metabolic networks participate in almost all the sets of the metabolic reactions from plants studied here. This is shown in Fig 3, where it can be observed that the proportion of metabolic reactions containing at least 1 metabolite belonging to the central core of each plant is much higher than the number of reactions that do not present it. Therefore, the results show that all the metabolic pathways are integrated with the most basal metabolism.

**Fig 4:**
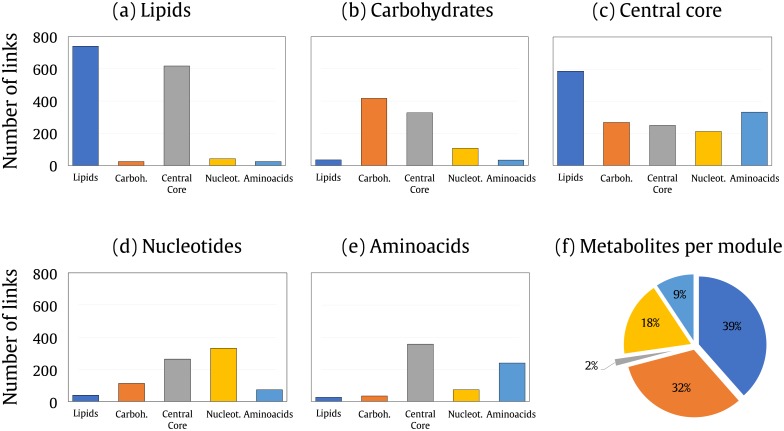
(a)-(e) Cross connection of metabolites from *Arabidopsis thaliana* clustered by metabolic modules: Lipids, Carbohydrates, *k*core central (core), Nucleotides and Amino acids. Each plot shows the number of links from metabolites, belonging to a respective module that are connected to metabolites from other modules. (f) Proportion of metabolites per modules relative to the full content of metabolites analyzed.

In earlier studies, it was revealed that metabolic networks of living beings have a modular and hierarchical structure [2]. In order to assess the levels of modularization of plant metabolic networks, we evaluated the connectivities of metabolic classes (modules) within the maximum *k*core to the metabolic network from *Arabidopsis thaliana* (see Fig 4). Nodes contained in the central layer that belong to any one of the addressed classes were removed from their respective classes and grouped in the maximum *k*core class in order to avoid ambiguity between the classes (see Material and methods section).

The graphs in Fig 4 demonstrate the high level of modularization of the metabolic network of *Arabidopsis thaliana* and also the high connectivity of the modules (metabolic classes) with the maximum *k*core of the network. It can be seen for example in Fig 4a that the lipids connect to other lipids and also to the central core in a much higher proportion than the other metabolic classes. It can also be observed in Fig 4c that the metabolites contained in the most central layer of the network are connected to the other metabolic classes at a higher proportion in all classes, which shows the enormous connectivity with the central layer. Therefore, it is clear that the network is modularized and centralizes the connections of the modules with the most central layer (maximum *k*core).

There are differences in the number of the metabolites in each class shown in Fig 4a to 4e. The proportion of metabolites from each class relative to the full content of metabolites in all classes is demonstrated in Fig 4f. The number of connections from each class is absolute, therefore the level of centralization in the central core is very high. For example the maximum *k*core class contains only 21 metabolites while the lipids contain around 500. This is almost 20 times higher than the content of the metabolites from the maximum *k*core, while the number of the links at the maximum *k*core is almost the same as the lipids.

### Differential distribution of the metabolism over the *k*core percolation layers

**Fig 5:**
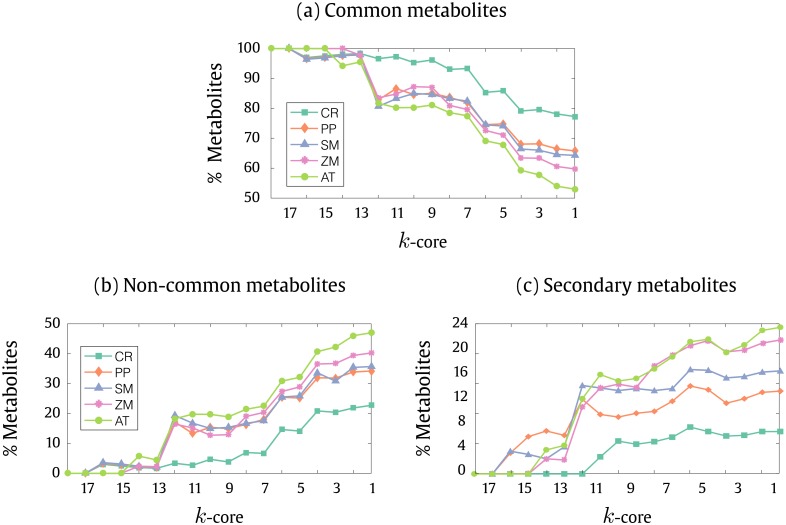
(a) Percentage of common metabolites shared with full content from each *k*core distributed along the percolation layers of the plant metabolic networks from Chlamydomonas reinhardtii, (CR) Physicomitrela patens, (PP) Selaginella moelendorffi, (SM) Zea mays(ZM) and Arabidopsis thaliana(AT). (b) Percentage of non-common metabolites distribution along the *k*layers. (c) Proportion of secondary metabolites shared with full content of the metabolites from each *k*core percolation level from the same 5 plants shown in (a).

We analyzed the proportion of common metabolites against the full content of metabolites across each *k*layer. These metabolites were extracted from the intersection dataset between the 17 plants studied in this work. The Fig 5a, shows the distribution of common metabolites across the *k*layers for plants belonging to five major clades, namely: the algae *Chlamydomonas reinhardtii*, the bryophyta moss *Physicomitrela patens*, the lycophyte *Selaginella moelendorffi* and two angiosperms, the monocotyledon *Zea mays* and the dicotyledon *Arabidopsis thaliana*. The analysis of the proportion from common metabolites in the *k*core layers reveals that its content is centered in the innermost *k*core, where these metabolites reach to almost the total content of the layers. The distribution of the metabolites per each *k*core layer for plants that were not shown in Fig 5 can be found in S1 File. On the other hand, the distribution of the complementary set to the common metabolism, the non-common metabolites between one plant and the other 16 plants studied, has an opposite decaying pattern along the *k*core layers, as it is a complementary set of the common metabolites, as shown in Fig 5b. Moreover, the previous analysis was repeated also to analyze the content of the secondary metabolites (see Fig 5c). Interestingly this distribution presented the same decaying pattern over the *k*layers as that shown to non-common metabolites between the plants (see Fig 5b).

This result reveals that the secondary metabolites are concentrated in the periphery of the networks and are absent in the inner layers. The distribution of the content of secondary metabolites along the *k*core layers is completely related to the non-common metabolic content between the plants. It is, therefore, possible that the distribution of the non-common metabolism throughout the *k*layers can be estimated as a reflection of the distribution of secondary metabolism.

At the same time, this analysis shows that the database of secondary metabolites, specifically cured for this work, can be estimated as a significant sample of the secondary metabolite content contained in PlantCyc. Its distribution over the *k*layers shows that the topological analysis of the network, by means of the *k*core algorithm, reveals information that correlates the topological structure of the metabolic network with the metabolic functionality, therefore discerning the basal and secondary metabolism according to their different topologies. Although algae, bryophytes and lycophytes show a much smaller amount of secondary metabolites in relation to the angiosperms, we observed that in the species of these clades, the secondary metabolites also have a peripheral location across the *k*cores.

It is important to emphasize that there are some secondary metabolites contained within the dataset of common metabolites found in the 17 plants. However, they are the minority and the analysis of the distribution of the non-common metabolites excludes the possibility of bias in our analysis of the distribution of the differential decay of the metabolism over the *k*layer. At the same time, it is interesting to notice that the simpler organism studied here is a unicellular algae *Chlamydomonas reinhardtii*, which already provides specialization of its metabolism and some secondary metabolites. Therefore, the intersection between the secondary and common metabolites among the 17 plants studied here probably derives from the fact that unicellular algae already present metabolic specialization. Additionally, it is also interesting to notice that the amount of secondary metabolites found at the dicotyledon *Arabidopsis thaliana* is greater than the ones found in the other plants (see Material and methods section and S1 File). Of course, as it is considered a model plant for the study of angiosperms, its molecular biology and biochemistry have been exhaustively studied, which logically encompasses its metabolism. This fact is clearly evidenced by the number of reactions available at the PlantCyc database. The results show that the basic general composition of the metabolism of any plant is centered in the innermost *k*core percolation layers and that metabolites belonging to these inner layers, with minor variations among the plants, are completely contained in the set of common metabolites of all plants. This also shows that the secondary metabolism is concentrated in the outer layers.

From Fig 5a and 5c, we can clearly observe an opposite relationship between secondary and common metabolites regarding the distribution of metabolites across the layers of the networks. This suggests that there may be a correlation between the metabolism specialization and the hierarchy established artificially by percolation in the *k*core layers.

This result supports the hypothesis that percolation in *k*cores provides information about the metabolism specialization and distribution of metabolites in complex networks associated with the topology. It also suggests the idea that the molecular evolution of metabolism can also be modeled by percolation in *k*cores.

It is known that the metabolism specialization along the plant evolution follows a pattern of descent with modification, with closely related species sharing more similar sets of metabolic reactions [9]. Therefore, it is also possible that the *k*core percolation reveals evolutionary information between plant species.

## Discussion

In this paper, we presented a model of the hierarchical structure of plant metabolic networks by means of the *k*core percolation layers, from which we observed the central core of 17 plant metabolic networks, reached by percolation. The metabolites found in this core were mainly the most basic components of the plant basal metabolism such as some ions used as electron transport (*H*^+^, $PO_{4}^{3-}$, $HCO_{3}^{-}$), metabolic currency (ATP, ADP, AMP), electron carriers (NADP+, NADPH, NAD, NADH), *H*_2_*O*, metabolites of the most basic energetic metabolism (pyruvate, oxaloacetate, Acetyl-Coa), amino acids (L-glutamate) and the most primary photosynthesis product, the oxygen (*O*_2_) (see Fig 2). This fact suggests that percolation generates some hierarchy of metabolic complexity along the formation of layers in the network.

The metabolite distribution across the plant’s metabolic reactions showed that at least one element of the set of metabolites belonging to the most central layer of the networks is present in more than 90% of the metabolic reactions from all plants studied here (see Fig 3). It is suggested that the central core elements can form a module that integrate all plant metabolic networks.

The analysis of the cross connection between metabolic classes showed that the metabolic classes in the *Arabidopsis thaliana* are highly connected within themselves, as the number of connections inside each metabolic class (Lipids, Carbohydrates, Nucleotides, and Amino acids) is higher than with other classes. Interestingly all classes are very connected with metabolites present at the maximum *k*core layer. This shows that in addition to being very modularized, the network is also very centralized in the metabolites found in the last level of the network percolation. Earlier studies have demonstrated that the metabolic networks have a modular and hierarchical structure [2].

It is also interesting to note that the common metabolism, represented by common metabolites between all plants studied here, is centralized in the *k*cores. Moreover, it is known that the basal metabolism arose early in the evolution of species, therefore it is not unreasonable to assume that the percolation by *k*cores provides a tool for studying molecular evolution metabolism, which is supposed to occur with the direction of the center to the periphery of the network percolation layers.

The peripheral location of the secondary and non-common metabolites between plants along the *k*core layers also corroborates the hypothesis that the *k*core percolation algorithm may reveal evolutionary hierarchies in the metabolism and it is appropriate to demonstrate connectivity hierarchies in plant metabolic networks and their correlation with the metabolic functionality.

It should be noted that the secondary metabolism provides alternative ways of molecular interactions between the plant and its environment and for this reason it appeared later in the evolution of species. This metabolism has a differential evolution in relation to the basal metabolism and genes related to it proliferate and diverge more rapidly throughout the evolution [9].

Regarding the curated dataset of secondary metabolites detailed in Material and Methods section, we must clarify that there is not an absolute distinction between basal and secondary metabolites, however, in general, it is meant by basal metabolism the set of metabolic reactions and metabolites that form the basic framework of metabolic pathways in common among all plants, from algae to dicotyledons. Besides the list of curated secondary metabolites, we have chosen to bring an additional information which refers to the distribution of non-common metabolites between plants within the framework of the *k*core percolation. We observed that the distribution of these metabolites is very similar to that of the secondary metabolites (see Fig 5). This fact was predictable and shows that the non-common metabolism between plants reflects the secondary metabolism. At the same time, this figure shows that, in fact, the secondary metabolism has a peripheral distribution in the layers of the network. In addition, it shows that the information generated in the database is pertinent and reveals topological information about plant metabolic networks.

The topological structure of the metabolic pathways along the *k*core layers can be assembled as a huge puzzle. Therefore, the logic of the formation of pathways may be elucidated through tracking pathway reactions along the *k*core percolation layers in different plant networks. Based on this, a new question can be put forward: How are the parts of the paths grouped to form a complete path along the *k*cores? The answer to this puzzle may simultaneously be how the molecular evolution of these routes is built in the various varieties of plants.

The *k*core percolation can also be useful to explain the operation of the routes in terms of the centralization of distribution of material (metabolites) along the topology of the networks, as the vast majority of the reactions contain at least one element of the central layer and therefore, appear to be embedded in the central module. This suggests that transport of matter at the metabolic pathways does not have an occasional structure and it can be centered in terms of routes. Moreover, it can be assumed that transport is performed by a chemical network, which although is not spatially defined, has logical connections that distribute material components (molecules). Therefore, theoretical correlations about the genesis and dynamics of pathways can be drawn from the knowledge of key metabolic nodes, which could be shown by metabolomic and transcriptomic data, for example. Therefore the *k*core analysis can reveal the hierarchy and emergence of these nodes in various topological states of the metabolic network.

All elements of the central core are considered hubs in plant metabolic networks. These metabolites are the basic bricks of which all metabolic pathways are built. Meanwhile, we observed that the metabolites at the maximum *k*core also participate in almost all chemical reactions of the secondary metabolism. This observation leads to proposing a model in which the *k*core central layer is a kind of network processing unit, which is responsible for anchoring the plant metabolic reactions.

Hierarchical network models show that their assembly hierarchy is possibly associated with the specialization of metabolism [2]. At the same time, our results reveal that the basal metabolism has strongly connected network nodes (metabolites) and the secondary metabolism has a more peripheral location across the *k*core percolation layers. It suggests that studies on the modularization of metabolism could be performed with *k*core percolation to reveals hierarchies of modularity in metabolism.

Finally, some questions remain: Can we assume that the molecular evolution of metabolism, i.e. adding and integrating new components can be simulated by the percolation proposed in this study? Is this addition anchored to the metabolites of the central layers?

## Material and methods

### Metabolic reaction database

The plant metabolic networks studied here were constructed from the metabolic reactions of 17 plants available at PlantCyc database (Version 9.5) [16], further details on how this database was constructed can be obtained in Ref. [19]. This database contains the reconstruction of the plant biochemical pathways based on an enzyme sequence from annotated metabolic functions of protein sequences [19–24]. This knowledge has allowed researchers to establish databases that are a good approximation of the content of plant metabolism and metabolic pathways. Although it can not be stated that the content of the reactions available in PlantCyc database is complete, it certainly reflects the annotation of much of the universe of known metabolic reactions and of their conference according to the annotated reactions based on gene sequences from enzymes. Certainly, until now, it is not known how many metabolic reactions can still be found at any plant through metabolomic science [25]. However metabolic reactions from plants with genome sequenced have been annotated based on DNA sequence information from known enzymes assigned according to reactions discovered. More details about how close are the data from metabolic reaction datasets relative to the complete annotation of metabolomes can be found in Ref. [25].

Table 1 shows the corresponding web references of the reaction datasets used in this work. This dataset provides information for the development of new hypotheses about the evolution and specialization of the plant metabolism [9]. Some hypotheses created from the available data were also used to develop our model.

**Table 1:** List of websites for metabolic reaction sets of the 17 plants studied in this work. These lists can be found in the Plantcyc database (version 9.5) [16].

Plant	Database of reactions
*Brachypodium distachyon*	https://pmn.plantcyc.org/BRACHYPODIUM/class-instances?object=Reactions
*Hordeum vulgare*	https://pmn.plantcyc.org/BARLEY/class-instances?object=Reactions
*Oryza sativa japonica*	https://pmn.plantcyc.org/ORYZA/class-instances?object=Reactions
*Panicum virgatum*	https://pmn.plantcyc.org/SWITCHGRASS/class-instances?object=Reactions
*Setaria italica*	https://pmn.plantcyc.org/SETARIA/class-instances?object=Reactions
*Sorghum bicolor*	https://pmn.plantcyc.org/SORGHUMBICOLOR/class-instances?object=Reactions
*Zea mays*	https://pmn.plantcyc.org/CORN/class-instances?object=Reactions
*Arabidopsis thaliana col*	https://pmn.plantcyc.org/ARA/class-instances?object=Reactions
*Brassica rapa pekinensis*	https://pmn.plantcyc.org/CHINESECABBAGE/class-instances?object=Reactions
*Carica papaya*	https://pmn.plantcyc.org/PAPAYA/class-instances?object=Reactions
*Glycine max*	https://pmn.plantcyc.org/SOY/class-instances?object=Reactions
*Manihot esculenta*	https://pmn.plantcyc.org/CASSAVA/class-instances?object=Reactions
*Populus trichocarpa*	https://pmn.plantcyc.org/POPLAR/class-instances?object=Reactions
*Vitis vinifera*	https://pmn.plantcyc.org/GRAPE/class-instances?object=Reactions
*Selaginella moellendorffii*	https://pmn.plantcyc.org/SELAGINELLA/class-instances?object=Reactions
*Physcomitrella patens*	https://pmn.plantcyc.org/MOSS/class-instances?object=Reactions
*Chlamydomonas reinhardtii*	https://pmn.plantcyc.org/CHLAMY/class-instances?object=Reactions

### Datasets

- *Common metabolites* This dataset is composed by the intersection among all metabolites of plants analyzed here. The dataset is available in S2 File.
- *Non-common metabolites* This dataset is complementary to the one described above, i.e. it is composed by those elements that do not belong to the intersection of metabolites among all the plants studied.
- *Secondary metabolites* This dataset was obtained by discriminating the complete dataset of metabolites of each plant. The secondary metabolites were identified using well-established literature regarding secondary metabolism [26]. The number of curated secondary metabolites per plant is shown in Table 2. The PlantCyc compound database [16] and the PubChem research tool [27] were used as a complementary resource to curate the list of secondary metabolites by comparing each metabolite against these databases. Although these databases may be partial in the classification between secondary and basal metabolism, their information is complementary (data available in S3 File).
- *Metabolic classes* This data set was obtained from the attribution of metabolites belonging to the classes: amino acids, nucleotides, lipids, carbohydrates and those metabolites found in the maximum *k*core of the metabolic network of the plant *Arabidopsis thaliana* (data available in S4 File). The metabolites from the maximum *k*core module that eventually belongs to the other modules were withdrawn from this class and reclassified as the maximum *k*core metabolite to avoid ambiguity.
- *Metabolites per kcore layer* The metabolite content for each plant metabolic network, distributed across *k*core percolation layers, was obtained by listing the metabolites during each level of the network percolation with the *k*core algorithm (data available in S5 File).

**Table 2:** Number of secondary metabolites per plant.

Plant	num. metabolites	Plant	#	Plant	#
BD	633	ZM	668	PT	732
HV	619	AT	824	VV	695
OSJ	691	BRP	750	SM	476
PV	690	CP	721	PP	373
SI	648	GM	706	CR	163
SB	645	ME	706		

### Metabolic network construction

We considered metabolic networks following the substrate-product network model [17]. The set of metabolic reactions from each plant was reduced into a metabolic network represented by an undirected graph consisting of a set of *N* metabolites.

The plant metabolic networks were modeled using the set of biochemical reactions from a specific plant. The set of metabolic networks used here are compiled and available in S6 File. Fig 1a and 1b) shows an example of the metabolic network modeling. For the sake of illustration, the plot shows two metabolic reactions that correspond to the network modeling in Fig 1b. In order to build the metabolic network, each metabolite reaction was considered. Thus, each metabolite is individually linked to each other with respect to the reaction arrows. Therefore, repeated metabolites and/or edges are dismissed. The metabolic network is represented by the adjacency matrix *A* of *N* × *N*, where each element *a*_*ij*_ is related with each other by a link.

### *k*core decomposition algorithm

The *k*core decomposition is an algorithm that splits a network into hierarchically ordered sub-structures (see S7 File). A *k*core layer is the maximal subgraph obtained by recursively removing all nodes with a degree lower than *k* until all nodes in the remaining graph have a degree larger than or equal to *k* [11, 12]. As this algorithm is an iterative procedure, it should not be confused with pruning nodes of a certain degree [15]. Fig 1b shows the topological percolation process in which the *k*cores are obtained from a given network. The node *i* in this network has a layer index *k* if it belongs to the *k*core but not to the (*k* + 1)-core. A *k*shell *G*_*k*_ is composed by all the nodes whose shell index is *k* [15]. The maximum value *k* in a given *G*_*k*_ is denoted by *k*_max_ [15].

### Cross connection of the metabolic modules

Metabolites belonging to the classes amino acids, nucleotides, lipids, carbohydrates and maximum *k*core were discriminated by identifying metabolites from each respective class present within the full content of metabolites from *Arabidopsis thaliana*.

The quantification of the cross connection of metabolites was performed by counting the number of links between metabolites belonging to a specific module with metabolites from other modules.

## Supporting information

S1 File Properties from the 17 plant metabolic networks. (PDF)

S2 File List of common metabolites. (XLSX)

S3 File List secondary metabolites. (XLSX)

S4 File List of metabolites separated by classes or modules used for the cross connection analysis. (XLSX)

S5 File List of metabolites per each layer. (XLSX)

S6 File Plant metabolic networks of the 17 plants species in GML format. (ZIP)

S7 File Supplementary video. A video corresponding to the *k*core decomposition for *Arabidopsis thaliana* metabolic network (Fig 1d). The video shows the decomposition process of the original network, layer by layer, until the maximum core is achieved. Each circle represents a metabolic node. The colors represent the layer for which the nodes belong to.
(MOV)
