# Supplementary material for: A hierarchical model of metabolic machinery based on the kcore decomposition of plant metabolic networks
Source: PLoS One. 2018 May 7;13(5):e0195843. doi: 10.1371/journal.pone.0195843 (PMC5937743; doi:10.1371/journal.pone.0195843)
Supplement: S1 File — (PDF) [file pone.0195843.s001.pdf]

Supplementary Information

A hierarchical model of metabolic machinery based on the $k$core decomposition of plant metabolic networks

Humberto A. Filho[1], Jeaneth Machicao[1], and Odemir M. Bruno[1,2]

[1]Scientific Computing Group. São Carlos Institute of Physics, University of São Paulo, São Carlos - SP, PO Box 369, 13560-970, Brazil.
[2]Corresponding author bruno@ifsc.usp.br

Properties from the 17 plant metabolic networks

Table S1: Properties from the 17 plant metabolic networks. The plants are classified according with their respective clade (first column) and species (second column). The plant acronym is given at third column. The number of reactions and metabolites (N) per plant is at fourth and fifth columns and the number of $k$core percolation layers $k_{\max}$ is at the last column.

Class	Plant	Reactions	N	$k_{\max}$
Monocotyledons	*BrachypodiumDistachyon*	2855	3107	16
	HordeumVulgare	2837	3059	16
	OryzaSativaJaponica	2936	3178	16
	PanicumVirgatum	2920	3165	17
	SetariaItalica	2878	3111	16
	SorghumBicolor	2877	3108	16
	ZeaMays	2898	3146	17
Dicotyledons	*ArabidopsisThalianaCol*	3424	3546	18
	BrassicaRapaPekinensis	3041	3249	17
	CaricaPapaya	2935	3155	17
	GlycineMax	3041	3242	17
	ManihotEsculenta	2991	3210	17
	PopulusTrichocarpa	3056	3274	16
	VitisVinifera	2954	3197	17
Lycophytes	*SelaginellaMoellendorffii*	2675	2924	17
Bryophytes	*PhyscomitrellaPatens*	2651	2858	18
Chlorophytes	*ChlamydomonasReinhardtii*	2208	2433	17

Supplementary Figure 1

	k_{18}	k_{17}	k_{16}	k_{15}	k_{14}	k_{13}	k_{12}	k_{11}	k_{10}	k_9	k_8	k_7	k_6	k_5	k_4	k_3	k_2	k_1
BD			100.0	100.0	97.6	98.0	84.6	87.4	85.6	86.3	82.2	81.1	72.8	72.0	63.8	63.6	61.2	60.5
HV			100.0	100.0	97.6	98.2	84.0	86.9	86.4	86.2	81.5	80.7	72.6	71.6	64.0	64.1	62.2	61.5
OSJ			100.0	100.0	100.0	100.0	84.9	85.5	83.6	85.0	78.8	78.9	71.1	69.9	62.7	62.5	59.8	59.2
PV		100.0	100.0	100.0	100.0	100.0	85.4	81.1	83.7	85.1	78.8	79.1	70.5	69.3	62.5	62.5	60.0	59.4
SI			100.0	100.0	97.7	98.2	83.3	83.3	82.6	84.0	78.8	78.5	71.7	70.8	63.5	63.4	61.0	60.4
SB			100.0	100.0	100.0	100.0	85.6	84.7	83.7	85.4	80.8	79.7	72.5	71.2	63.9	63.7	61.2	60.5
ZM		100.0	100.0	100.0	97.6	97.8	83.5	84.8	87.3	87.1	81.0	79.6	72.6	71.1	63.4	63.3	60.6	59.8
AT	100.0	100.0	100.0	100.0	94.2	95.5	81.7	80.2	80.3	81.1	78.6	77.5	69.2	67.9	59.3	57.8	54.0	53.0
BRP		100.0	100.0	100.0	97.6	97.9	85.4	86.6	81.7	81.7	77.0	76.0	70.5	69.6	63.1	62.1	58.6	57.8
CP		100.0	100.0	100.0	97.7	97.9	81.7	82.6	83.9	83.6	79.9	78.1	69.9	68.7	64.1	63.4	60.4	59.6
GM		100.0	100.0	100.0	97.6	98.2	83.5	83.6	83.7	85.2	80.4	78.6	68.7	67.2	61.3	61.3	58.6	58.0
ME		100.0	100.0	100.0	97.7	98.1	82.7	83.1	84.7	83.6	76.5	75.6	68.4	67.7	61.7	61.8	59.2	58.6
PT			100.0	100.0	97.7	98.2	83.5	84.6	83.6	83.4	75.7	74.9	67.4	66.7	60.9	61.0	58.1	57.4
VV		100.0	100.0	100.0	97.7	98.1	81.3	81.5	79.4	81.1	75.6	75.4	69.1	68.8	62.3	62.4	59.5	58.8
SM		100.0	96.4	96.9	97.6	97.9	80.6	83.2	85.0	84.6	83.3	82.4	74.5	74.1	66.4	66.7	64.6	64.3
PP	100.0	100.0	96.7	97.1	97.7	98.0	82.8	86.6	84.6	85.1	83.6	81.9	74.6	74.8	68.1	68.1	66.1	65.8
CR		100.0	97.0	97.6	98.0	98.3	96.6	97.3	95.3	96.2	93.0	93.3	85.3	85.9	79.1	79.6	78.1	77.3

Figure S1: Heatmap of the proportion (percentage) of metabolites from each $k$core layer inside the set of common metabolites of the plants described according to the acronyms. Distribution from the most external $k = 1$, until the most central core in all plant networks.

Supplementary Figure 2

	k_{18}	k_{17}	k_{16}	k_{15}	k_{14}	k_{13}	k_{12}	k_{11}	k_{10}	k_9	k_8	k_7	k_6	k_5	k_4	k_3	k_2	k_1
BD			0.0	0.0	2.4	2.0	12.1	10.9	15.3	14.8	16.1	17.2	18.8	19.2	18.8	19.2	19.8	20.4
HV			0.0	0.0	2.4	5.4	12.8	11.5	11.8	13.8	16.1	16.8	19.3	20.1	19.1	19.5	19.8	20.2
OSJ			0.0	0.0	0.0	0.0	11.6	13.7	17.7	16.4	19.3	19.3	20.2	21.2	20.0	20.3	21.4	21.7
PV		0.0	0.0	0.0	0.0	0.0	11.2	18.2	17.7	16.4	19.1	18.8	20.4	21.6	20.2	20.4	21.4	21.8
SI			0.0	0.0	2.3	5.4	11.5	14.3	16.0	14.9	18.4	18.5	19.9	20.7	19.3	19.6	20.4	20.8
SB			0.0	0.0	0.0	3.8	11.1	14.5	15.8	13.9	16.2	17.4	19.4	20.6	19.1	19.4	20.2	20.8
ZM		0.0	0.0	0.0	2.4	2.2	10.6	13.6	14.2	13.7	17.1	18.8	20.3	21.1	19.3	19.6	20.7	21.2
AT	0.0	0.0	0.0	0.0	3.8	4.5	11.9	15.7	14.7	15.1	16.6	18.5	20.9	21.3	19.2	20.4	22.7	23.2
BRP		0.0	0.0	0.0	2.4	4.3	10.1	11.8	11.9	13.5	16.6	18.3	19.9	20.7	19.0	20.5	22.3	23.1
CP		0.0	0.0	0.0	2.3	2.1	9.7	12.2	13.5	14.9	16.2	18.3	20.4	21.6	19.9	20.8	22.0	22.9
GM		0.0	0.0	0.0	2.4	5.4	11.3	14.2	15.0	13.8	16.9	18.5	21.6	22.7	20.3	20.4	21.5	21.8
ME		0.0	0.0	0.0	2.3	5.8	12.2	14.5	14.9	15.7	21.4	22.4	22.7	23.0	20.6	20.7	21.6	22.0
PT			0.0	0.0	2.3	5.5	11.3	13.1	13.7	15.2	20.5	21.8	22.4	22.9	20.4	20.6	21.9	22.4
VV		0.0	0.0	0.0	2.3	5.7	10.4	14.1	14.7	14.6	18.4	19.1	21.4	21.8	20.3	20.6	21.3	21.7
SM		0.0	3.6	3.1	2.4	4.3	14.0	13.6	13.2	13.5	13.2	13.5	16.5	16.4	15.2	15.4	16.1	16.3
PP	0.0	0.0	3.3	5.9	6.8	6.1	11.8	9.4	9.0	9.6	9.9	11.5	13.9	13.3	11.2	11.9	12.9	13.1
CR		0.0	0.0	0.0	0.0	0.0	0.0	2.7	5.2	4.7	5.1	5.8	7.4	6.7	6.0	6.1	6.7	6.7

Figure S2: Heatmap indicating the proportion (percentage) of secondary metabolites contained in each $k$core network layer. Plants are indicated by their acronyms in the first column of the table and $k$cores numbers are indicated by k.
